# Prognostic Value of the Leuko-Glycaemic Index in the Postoperative Period of Coronary Artery Bypass Grafting

**DOI:** 10.21470/1678-9741-2020-0349

**Published:** 2021

**Authors:** Leonardo Adrián Seoane, Lucrecia Burgos, Juan Carlos Espinoza, Juan Francisco Furmento, Mariano Noel Benzadón, Juan Mariano Vrancic, Fernando Piccinini, Daniel Navia

**Affiliations:** 1 Department of Critical Care Cardiology, Instituto Cardiovascular de Buenos Aires (ICBA), Buenos Aires, Argentina.; 2 Department of Heart Failure, Instituto Cardiovascular de Buenos Aires (ICBA), Buenos Aires, Argentina.; 3 Department of Cardiovascular Surgery, Instituto Cardiovascular de Buenos Aires (ICBA), Buenos Aires, Argentina.

**Keywords:** Glycemic Index. Cardiac Surgical Procedures. Postoperative Period. Leukocytes. Critical Care. Cardiac Output, Low. Aged

## Abstract

**Introduction:**

High leuko-glycaemic index (LGI) (> 2000) has been associated with poor prognosis in many critical care settings. However, there is no evidence of LGI’s prognostic value in the postoperative period of coronary artery bypass grafting (CABG). This study aims to analyze the prognostic value of LGI in the postoperative period of CABG.

**Methods:**

Single-center retrospective analysis of prospectively collected data was performed. Consecutive adult patients undergoing CABG between 2007 and 2019 were included. Blood glucose levels and white blood cells count were evaluated in the immediate postoperative period. LGI was calculated by multiplying both values and dividing them by 1,000 and analyzed in quartiles. Receiver operating characteristic curve was used to determine the best cutoff value. The primary combined endpoint was in-hospital mortality, low cardiac output (LCO), or acute kidney injury (AKI). Secondary endpoints included in-hospital death, AKI, atrial fibrillation, and LCO.

**Results:**

The study evaluated 3,813 patients undergoing CABG (88.5% male, 89.8% off-pump surgery, aged 64.6 years [standard deviation 9.6]). The median of LGI was 2,035. Presence of primary endpoint significantly increased per LGI quartile (9.2%, 9.7%, 11.8%, and 15%; *P*<0.001). High LGI was associated with increased occurrence of in-hospital mortality, LCO, AKI, and atrial fibrillation. The best prognostic cutoff value for primary endpoint was 2,000. In a multivariate logistic regression model, high LGI was independently associated with in-hospital death, LCO, or AKI.

**Conclusion:**

High LGI was an independent predictor of in-hospital mortality, LCO, or AKI in postoperative period of CABG. It was also associated with higher in-hospital death.

**Table:** 

Abbreviations, acronyms & symbols				
AF	= Atrial fibrillation		LGI	= Leuko-glycaemic index
AKI	= Acute kidney injury		LITA	= Left internal thoracic artery
AMI	= Acute myocardial infarction		OR	= Odds ratios
CABG	= Coronary artery bypass grafting		PCI	= Percutaneous coronary intervention
CI	= Confidence intervals		Q	= Quartile
COPD	= Chronic obstructive pulmonary disease		RITA	= Right internal thoracic artery
EuroSCORE	= European System for Cardiac Operative Risk Evaluation		ROC	= Receiver operating characteristic
IQR	= Interquartile range		SD	= Standard deviation
LCO	= Low cardiac output		STS	= Society of Thoracic Surgeons

## INTRODUCTION

Currently, stratification of surgical risk in cardiac surgery is mainly evaluated through different risk scoring systems, such as the European System for Cardiac Operative Risk Evaluation (EuroSCORE) and the Society of Thoracic Surgeons (STS) scores ^[^^1^^-^^3^^]^. However, these scores use variables collected preoperatively and do not consider multiple prognostic variables that are important in the early postoperative period. Scoring systems that are commonly used in general intensive care units, such as the Sequential Organ Failure Assessment and the Acute Physiology and Chronic Health Evaluation II, may be an alternative for determining the risk of patients in cardiac surgical care units ^[^^4^^,^^5^^]^. Nevertheless, most of risk scores were not validated in special units. Specifically, cardiac surgery patients have been excluded from the development studies of general predictive scoring systems ^[^^6^^]^.

In relation to postoperative biomarkers, lactic acid and mixed venous oxygen saturation are classic predictors with prognostic value in patients undergoing cardiac surgery ^[^^7^^-^^10^^]^. However, these isolated markers fail to predict the real patient’s risk. Hyperglycemia in the immediate postoperative period is also associated with increased morbidity and mortality ^[^^11^^]^. In addition, there is evidence that after the use of cardiopulmonary bypass in cardiac surgery, plasma concentration of leukocyte mediators increases, contributing to the systemic inflammatory response syndrome and worse prognosis ^[^^12^^,^^13^^]^.

The leuko-glycaemic index (LGI) is an index that combines white blood cell count and blood glucose, calculated by multiplying both values and dividing them by a thousand. This index could be a marker of systemic inflammatory response syndrome and has already shown to be a good predictor of events in various scenarios of critical care medicine, such as acute myocardial infarction and stroke ^[^^14^^-^^19^^]^. Seoane et al. ^[^^20^^]^ have demonstrated its prognostic value for the first time in cardiac surgery in 2017. However, LGI was evaluated in a heterogeneous population since it included different types of cardiac surgeries. Furthermore, high LGI was not associated with higher in-hospital death.

The aim of this study was to analyze the prognostic value of LGI in the postoperative period of coronary artery bypass grafting (CABG).

## METHODS

### Study Design

This is a single-center retrospective observational study. Data were collected retrospectively from our computerized custom-made database (Microsoft Access; Microsoft Corp, Redmond, WA), which collects prospective information on cardiac surgery procedures and is used daily for clinical data management. The institutional ethical committee approved the trial, and surgical consent was obtained from each patient with respect to surgical method and postoperative evaluations. The study included consecutive patients undergoing CABG in the Instituto Cardiovascular de Buenos Aires between January 2007 and September 2019. Patients who underwent cardiac surgery other than CABG were excluded.

### Leuko-Glycaemic Index

Blood samples were collected into serum separator tubes in the immediate postoperative period, upon admission to the intensive care unit. Blood glucose levels were expressed in mg/dl, and white blood cells count in cells per mm^3^. LGI was calculated by multiplying both values and dividing them by a thousand. LGI units were expressed in mg/dl. mm^3^.

These samples were selected in the immediate postoperative period and as the only values, since they were used in the main studies of LGI in other clinical settings, in which the admission values to the critical care unit were considered. Furthermore, due to the standardized management in the operating room, this unique LGI would not have variations derived from different postoperative complications that could occur in the recovery unit.

### Cardiac Surgery and Operating Room Management

CABG procedures were most of them off-pump surgeries. The criteria used for conversion to on-pump CABG were hemodynamic or electric instability and calcified and intramyocardial coronary arteries. The surgical technique used for CABG procedure consisted in use both internal thoracic arteries (left internal thoracic artery [LITA] and right internal thoracic artery [RITA]) as main conduits for coronary revascularization. If necessary, saphenous vein grafts were used. Most internal thoracic artery grafts were harvested in skeletonized fashion and the most commonly technical configuration used was in-situ anastomoses of LITA to the left anterior descending coronary artery, and RITA, after being divided at its origin, was connected end-to-side to the in-situ LITA as a sequential T graft to the circumflex and to the posterior descending coronary artery. Elective and emergency procedures were included.

Anesthetic, surgical, and postoperative management were broadly the same for all patients. Anesthesia was induced with a combination of fentanyl (10 to 15 mcg/kg), propofol (0.5 to 2 mg/kg), and pancuronium (0.1 mg/kg). Antimicrobial prophylaxis was provided with amikacin and vancomycin, according to institutional protocol. Anesthesia was maintained during the surgery with volatile anesthetic agents. In the operating room, patients received intravenous insulin according to the STS practice guideline series ^[^^21^^]^. Glucose levels > 180 mg/dL were treated initially with a single or intermittent dose of intravenous insulin. In those patients who had persistently elevated serum glucose (>180 mg/dl), an intravenous insulin infusion was initiated to maintain serum glucose < 180 mg/dl.

### Study Endpoints

The primary combined endpoint was in-hospital mortality, low cardiac output (LCO), or acute kidney injury (AKI).

LCO was defined by pulmonary catheter thermodilution as cardiac index < 2.2 liters/min/m^2^, associated with wedge pressure > 18 mmHg, hypoperfusion, and vasopressor drug requirement.

AKI was defined by the Kidney Disease: Improving Global Outcomes score, as an increase in serum creatinine greater than two times from baseline or urine output < 0.5 ml/kg/hour for more than 12 hours.

The primary endpoint was evaluated as a combined outcome. In contrast, secondary endpoints were individually computed. They included LCO, in-hospital mortality, AKI, and atrial fibrillation, which were analyzed separately.

Postoperative atrial fibrillation was defined as in previous studies, as any documented atrial fibrillation episode lasting > 30 seconds recorded either by continuous telemetry throughout hospitalization or on a 12-lead electrocardiogram performed daily and when the patient reported experiencing symptoms. All patients had continuous telemetry monitoring at least during the first 48 hours by an offsite central monitor unit, and once identified, every arrhythmia event was confirmed by a cardiologist.

### Statistical Analysis

Continuous variables were examined by Kolmogorov-Smirnov test to check for normality of distribution. According to distribution, continuous variables were presented as a mean ± standard deviation (SD) or a median and interquartile range (IQR). LGI was analyzed in quartiles according to 25, 50, and 75 percentile values. Student’s *t*-test and Mann-Whitney U tests were used to compare parametric and nonparametric continuous variables, respectively. Categorical data were presented as percentages or frequencies and were compared by Chi-square (v2) test. The receiver operating characteristic (ROC) curve was used to determine the best LGI cutoff value to predict the primary endpoint. Variables significantly associated with in-hospital death, LCO, or AKI after univariate analysis (*P*<0.05) were entered in a multivariable logistic regression model. The variables that were independently associated with the primary combined endpoint were presented as odds ratios (ORs), along with the 95% confidence intervals (CIs).

A two-tailed *P*-value < 0.05 was considered as statistically significant. All data were analyzed using IBM Corp. Released 2010, IBM SPSS Statistics for Windows, Version 19.0, Armonk, NY: IBM Corp.

### Ethical Considerations

This study was approved by the institution’s research and ethics board. The study was a retrospective investigation with de-identified data; according to national regulations, request for informed consent was waived. At the time of hospitalization, the patient signed consent for the transfer of personal data for scientific purposes.

## RESULTS

The study evaluated 3,813 patients undergoing CABG, most of them male (68.5%). The mean age was 64.6 (SD 9.6) years. Regarding cardiovascular risk factors, 77% had arterial hypertension and 29% had diabetes. In relation to the presence of other comorbidities, 41% had prior acute myocardial infarction, 9% had history of atrial fibrillation, 16% had severe left ventricular dysfunction, 5% had chronic obstructive pulmonary disease, and 5% had chronic renal failure. The mean EuroSCORE II was 3.6 (SD 2.4). In the population analysis, according to LGI quartiles, there was no difference in the baseline characteristics, except for the highest percentage of diabetes in the highest quartile (Table 1).

**Table 1:** Basal characteristics of the population according to LGI.

Variable	LGI quartiles				*P*-value
	Q1 (n=955)	Q2 (n=954)	Q3 (n=951)	Q4 (n=953)	
Sex, male, n (%)	648 (67.9)	668 (70.0)	649 (68.2)	632 (66.3)	0.084
Age, years (SD)	64.8 (9.6)	64.0 (9.5)	64.3 (9.5)	64.7 (9.8)	0.228
Weight, kg (SD)	79.3 (30.9)	81.5 (27.3)	83.0 (47.7)	80.6 (33.9)	0.144
Height, cm (SD)	163.8 (41.7)	164.3 (35.6)	163.9 (34.5)	162.6 (37.7)	0.764
Hypertension, n (%)	718 (75.2)	739 (77.5)	748 (78.7)	738 (77.4)	0.332
Smoking, n (%)	576 (60.3)	599 (62.8)	588 (61.8)	564 (59.2)	0.381
Diabetes, n (%)	243 (25.4)	225 (23.6)	259 (27.2)	351 (36.8)	< 0.001
Family history of heart disease, n (%)	191 (20.0)	208 (21.8)	230 (24.1)	226 (23.7)	0.119
Prior AMI, n (%)	396 (41.5)	391 (41.0)	396 (41.6)	394 (41.3)	0.993
Prior PCI, n (%)	213 (22.3)	211 (22.1)	192 (20.2)	179 (18.8)	0.182
Peripheral vascular disease, n (%)	58 (6.1)	46 (4.8)	43 (4.5)	53 (5.6)	0.208
Prior stroke, n (%)	35 (3.7)	40 (4.2)	29 (3.0)	32 (3.4)	0.578
Chronic kidney disease, n (%)	50 (5.2)	53 (5.6)	47 (4.9)	42 (4.4)	0.700
Atrial fibrillation, n (%)	99 (10.4)	77 (8.1)	91 (9.6)	84 (8.8)	0.245
Left ventricular dysfunction, n (%)	167 (17.5)	160 (16.8)	136 (14.3)	148 (15.5)	0.242
COPD, n (%)	52 (5.4)	63 (6.6)	45 (4.7)	42 (4.4)	0.145
EuroSCORE II, (SD)	3.7 (2.3)	3.4 (2.4)	3.6 (2.5)	3.7 (2.5)	0.628

AMI=acute myocardial infarction; COPD=chronic obstructive pulmonary disease; EuroSCORE=European System for Cardiac Operative Risk Evaluation; LGI=leuko-glycaemic index; PCI=percutaneous coronary intervention; Q=quartile; SD=standard deviation

CABG procedures were mainly off-pump (88.8%) and elective surgeries (64.1%). Urgent procedures and number of bypasses were similar in each quartile of the population. Nevertheless, the use of cardiopulmonary bypass was higher in quartile 4 (Table 2).

**Table 2:** Surgical and laboratory characteristics according to LGI.

Variable	Total (n=3813)	LGI quartiles				*P*-value
		Q1 (n=955)	Q2 (n=954)	Q3 (n=951)	Q4 (n=953)	
**CABG characteristics**						
Elective surgery, n (%)	2444 (64.1)	595 (62.3)	631 (66.1)	612 (64.3)	606 (63.6)	0.364
Cardiopulmonary bypass, n (%)	427 (11.2)	118 (12.3)	81 (8.5)	83 (8.7)	145 (15.2)	0.001
Number of bypasses, n (SD)	3.1 (0.7)	3.1 (0.7)	3.2 (0.7)	3.0 (0.7)	3.1 (0.7)	0.571
**Laboratory characteristics**						
Prior creatinine, mg/dl (SD)	1.09 (0.32)	1.11 (0.39)	1.10 (0.45)	1.09 (0.34)	1.08 (0.31)	0.615
Prior hematocrit, % (SD)	39.2 (4.1)	38.6 (4.4)	39.1 (4.6)	39.4 (4.3)	39.1 (5.0)	0.487
First-day postoperative blood glucose level, mg/dl (SD)	165.4 (25)	139.8 (27)	157.7 (24)	171.3 (29)	193.8 (33)	< 0.001
First-day postoperative white blood cells count, cell/mm^3^ (SD)	12262 (2146)	10240 (2486)	11744 (2097)	13810 (2483)	15171 (5739)	< 0.001
Postoperative lymphocyte/neutrophil ratio (SD)	11.7 (1.8)	5.4 (1.9)	9.6 (1.7)	13.8 (1.8)	18.5 (2.4)	< 0.001
Postoperative C-reactive protein, mg/L (SD)	51.6 (4.7)	31.1 (4.8)	45.6 (4.6)	57.2 (5.0)	63.8 (7.1)	< 0.001

CABG=coronary artery bypass grafting; LGI=leuko-glycaemic index; Q=quartile; SD=standard deviation

**Fig. 1:**
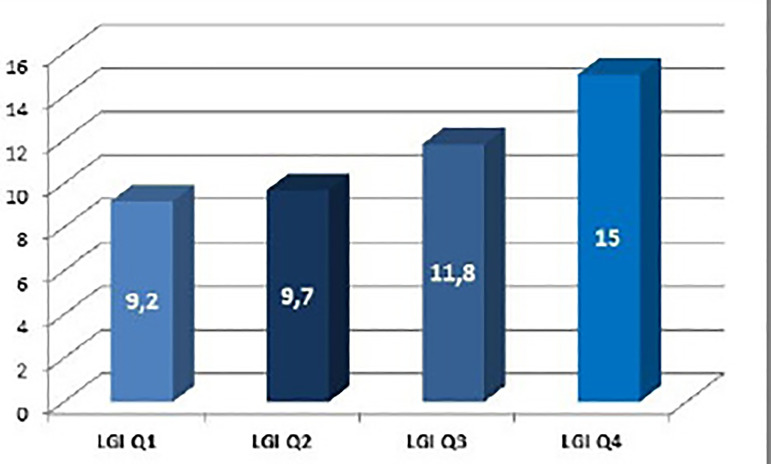
Incidence (%) of primary combined endpoint according to the leuko-glycaemic index (LGI). Q=quartile

The median LGI was 2035 mg/dl. mm^3^ (IQR 25-75%: 1641 - 2683). The rate of in-hospital mortality, LCO, or AKI was 11.4%. Presence of the primary endpoint significantly increased per LGI quartile (9.2%, 9.7%, 11.8%, and 15%; *P*<0.001) (Figure 1). The ROC curve value for this combined endpoint was 0.61 (95% CI 0.56 - 0.67). High LGI was associated with a statistically significant increased occurrence of in-hospital death, LCO, AKI, and postoperative atrial fibrillation (Table 3).

**Table 3:** Primary and secondary outcomes according to LGI.

Variable	Total n=3813	LGI quartiles				*P*-value
		Q1	Q2	Q3	Q4	
		(LGI: 0 – 1641) n=955	(LGI: 1642 – 2035) n=954	(LGI: 2036 – 2683) n=951	(LGI > 2683) n=953	
In-hospital death, LCO, or AKI, n (%)	436 (11.4)	88 (9.2)	93 (9.7)	112 (11.8)	143 (15.0)	< 0.001
In-hospital death, n (%)	59 (1.5)	11 (1.2)	11 (1.2)	13 (1.4)	24 (2.5)	0.045
LCO, n (%)	135 (3.5)	21 (2.2)	22 (2.3)	34 (3.6)	58 (6.1)	< 0.001
Postoperative AF, n (%)	507 (13.3)	108 (11.3)	125 (13.1)	120 (12.6)	154 (16.2)	0.015
Postoperative AKI, n (%)	317 (8.3)	58 (6.1)	71 (7.4)	92 (9.7)	96 (10.1)	0.004

AF=atrial fibrillation; AKI=acute kidney injury; LCO=low cardiac output; LGI=leuko-glycaemic index; Q=quartile

The best prognostic cutoff value for the primary endpoint obtained by ROC curve was 2000 mg/dl. mm^3^. In a multivariate logistic regression model, high LGI was independently associated with in-hospital death, LCO, or AKI (OR: 1.50, 95% CI 1.21 - 1.87; *P*<0.001) (Table 4).

**Table 4:** Multivariate logistic regression model for in-hospital mortality, LCO, or AKI.

Variables	OR	95% CI		*P*-value
LGI > 2000	1.508	1.213	1.874	< 0.001
Age (per year)	1.035	1.024	1.047	< 0.001
Female	1.342	1.011	1.782	0.042
Diabetes	1.446	1.168	1.791	0.001
Elective surgery	0.590	0.480	0.725	< 0.001
Severe left ventricular dysfunction	2.327	1.840	2.944	< 0.001
Cardiopulmonary bypass	2.334	1.690	3.238	< 0.001

AKI=acute kidney injury; CI=confidence interval; LCO=low cardiac output; LGI=leuko-glycaemic index; OR=odds ratio

## DISCUSSION

In this large cohort of patients, high LGI was an independent predictor of in-hospital mortality, LCO, or AKI in the postoperative period of CABG. It was also associated with higher in-hospital death.

Hyperglycemia is very common in the postoperative period of cardiac surgery, affecting approximately 33% of patients, whether they are diabetic or not ^[^^22^^]^. Furthermore, the immediate postoperative blood glucose was predictive of complications (prolonged in-hospital stay, higher infection rates, and increased postoperative morbidity), particularly in those patients with levels > 250 mg/dl causing a 10-fold increase in risk ^[^^23^^]^. Thereafter, Doenst et al. ^[^^11^^]^ showed that hyperglycemia during cardiopulmonary bypass was an independent risk factor for mortality in patients undergoing cardiac surgery.

Regardless of hyperglycemia, diabetic patients after cardiovascular surgery present higher perioperative morbidity and mortality. Insulin deficiency and insulin resistance are aggravated by surgery and anesthesia and may lead to lipolysis, metabolic acidosis, electrolyte changes, and increased protein catabolism. Insulin administration is high among CABG patients and could reverse most of these metabolic disorders. However, Ranney et al. ^[^^24^^]^ showed that insulin use did not change prognosis in diabetic patients and that it was associated with a higher risk of adverse outcomes in patients without diabetes.

Non-diabetic patients also frequently present hyperglycemia in the postoperative period of cardiovascular surgery, which is a risk factor for mortality. In these patients, preoperative screening with Homeostasis Model Assessment of Insulin Resistance might provide an insight to the glucometabolic state of individuals before CABG, and could be a predictive value for postoperative outcomes ^[^^25^^]^.

The metabolic stress of the surgery increases levels of glucagon, growth hormone, and cortisol and is enhanced by prolonged fasting and decreased insulin levels. Within this response to trauma, insulin resistance and hyperglycemia are manifested, being more intense in the first postoperative days and in major surgeries. In 2012, Feguri et al. ^[^^26^^]^ showed that preoperative fasting abbreviation with administration of liquid enriched with carbohydrates in CABG was safe and improved the glycemic control in the intensive care unit. Afterwards, the same author described that this strategy added to intraoperative infusion of Omega-3 polyunsaturated fatty acids supported faster postoperative recovery in patients undergoing on-pump CABG ^[^^27^^]^.

Leukocytosis is also a frequent finding in cardiac surgery. Inflammation is produced by humoral and cellular interactions with numerous pathways and multiple mediators ^[^^12^^,^^28^^]^. It is known that cardiopulmonary bypass induces a systemic inflammatory reaction, that may contribute to postoperative complications, including myocardial dysfunction, AKI, respiratory failure, bleeding disorders, altered liver function, and multiorgan failure ^[^^13^^,^^29^^]^. Neutrophil to lymphocyte ratio and platelet to lymphocyte ratio are clinical parameters, which can be found in a simple hemogram and increase in systemic inflammation. These ratios were independent biomarkers directly related to development of AKI in the early postoperative period of isolated CABG. Parlar et al. ^[^^30^^]^ showed that increased postoperative first-day leukocyte count was also associated with AKI in these patients.

LGI is an index that combines white blood cell count and blood glucose levels. It is calculated by multiplying both values and dividing them by a thousand. It was first described in 2010 by Quiroga Castro et al. ^[^^14^^]^ to determine prognostic value in patients with acute myocardial infarction. In that study, LGI > 1600 mg/dl. mm^3^ was associated with in-hospital complications (acute myocardial infarction, heart failure, and post-infarction angina). Hirschson Prado et al. ^[^^15^^]^ also showed that high LGI was an independent predictor of poor evolution in acute myocardial infarction (in hospital death or Killip Kimball 3-4). Although it was a novel prognostic index, it was evaluated in a small number of patients. Afterwards, León et al. ^[^^16^^]^ and Rodriguez Jimenez et al. ^[^^17^^]^ also proved that high LGI in the course of an acute myocardial infarction could be associated with a higher in-hospital death. In addition, Caldas et al. ^[^^18^^]^ and García Alvarez et al. ^[^^19^^]^ showed that LGI was a risk marker for predicting mortality in patients with ischemic stroke.

We previously demonstrated its prognostic value for the first time in cardiac surgery in 2017. In that study, a large number of patients were evaluated (n=2,743) and different types of cardiac surgeries were included ^[^^20^^]^. Although high LGI was associated with an increased occurrence of in-hospital death or LCO, a more heterogeneous population was evaluated. Furthermore, high LGI was not associated with higher in-hospital death or atrial fibrillation. In this study, more patients were evaluated (n=3,813), and only CABG surgeries were included, analyzing a more homogeneous population, mainly off-pump. Population characteristics and cutoff value of LGI for primary combined endpoint were similar in both studies. In contrast, in this study, high LGI predicted in-hospital death and atrial fibrillation as secondary events.

LGI, being determined by blood glucose levels and white blood cell count, could be a marker of systemic inflammation. Therefore, it is logically associated with poor outcomes in various scenarios of critical care medicine, including cardiac surgery. Even in this population with CABG mainly off-pump, in which systemic inflammatory reaction is lower, this index could predict in-hospital death, LCO, and AKI. Values of LGI > 2000 mg/dl. mm^3^ may be the best predictive values for the primary combined endpoint.

Concerning clinical relevance, LGI being a simple index available in any intensive care unit, easy to calculate, and low cost, could be a valuable prognostic tool to stratify patients in the immediate postoperative period of CABG. Those patients with high LGI values may benefit from greater monitoring and from using early therapeutic strategies to reduce LCO, AKI, and atrial fibrillation.

### Limitations

The present study has several potential limitations. First, it is a retrospective analysis of prospectively collected data, with the biases that this entails. Second, it was developed in a high-complexity cardiovascular center, which limits the generality of the findings. Third, there is a low proportion of women and rather young age of the population included in this study. Finally, another possible limitation is the high rate of off-pump surgery, which is not the main technique of CABG procedures worldwide.

## CONCLUSION

High LGI was an independent predictor of in-hospital mortality, LCO, or AKI in the postoperative period of CABG. It was also associated with a significant increased occurrence of in-hospital death, LCO, AKI, and atrial fibrillation. Due to these findings, the use of this simple and novel index could be an attractive tool to stratify risk in patients undergoing CABG.

**Table:** 

Authors' roles & responsibilities	
LAS	Substantial contributions to the conception or design of the work; or the acquisition, analysis or interpretation of data for the work; drafting the work or revising it critically for important intellectual content; agreement to be accountable for all aspects of the work in ensuring that issues related to the accuracy or integrity of any part of the work are appropriately investigated and resolved; final approval of the version to be published
LB	Substantial contributions to the conception or design of the work; or the acquisition, analysis or interpretation of data for the work; drafting the work or revising it critically for important intellectual content; agreement to be accountable for all aspects of the work in ensuring that issues related to the accuracy or integrity of any part of the work are appropriately investigated and resolved; final approval of the version to be published
JCE	Substantial contributions to the conception or design of the work; or the acquisition, analysis or interpretation of data for the work; drafting the work or revising it critically for important intellectual content; agreement to be accountable for all aspects of the work in ensuring that issues related to the accuracy or integrity of any part of the work are appropriately investigated and resolved; final approval of the version to be published
JFF	Substantial contributions to the conception or design of the work; or the acquisition, analysis or interpretation of data for the work; drafting the work or revising it critically for important intellectual content; agreement to be accountable for all aspects of the work in ensuring that issues related to the accuracy or integrity of any part of the work are appropriately investigated and resolved; final approval of the version to be published
MNB	Substantial contributions to the conception or design of the work; or the acquisition, analysis or interpretation of data for the work; drafting the work or revising it critically for important intellectual content; agreement to be accountable for all aspects of the work in ensuring that issues related to the accuracy or integrity of any part of the work are appropriately investigated and resolved; final approval of the version to be published
